# Candidate gene screening for lipid deposition using combined transcriptomic and proteomic data from Nanyang black pigs

**DOI:** 10.1186/s12864-021-07764-2

**Published:** 2021-06-12

**Authors:** Liyuan Wang, Yawen Zhang, Bo Zhang, Haian Zhong, Yunfeng Lu, Hao Zhang

**Affiliations:** 1 College of Life Science and Agricultural Engineering, Nanyang Normal University, Nanyang, China; 2 National Engineering Laboratory for Animal Breeding/Beijing Key Laboratory for Animal Genetic Improvement, China Agricultural University, Beijing, China; 3 Shenzhen Branch, Guangdong Laboratory for Lingnan Modern Agriculture, Agricultural Genomics Institute at Shenzhen, Chinese Academy of Agricultural Sciences, Shenzhen, China

**Keywords:** Genetic divergence, Lipid deposition, Multi-omics, Nanyang black pig, Phenotypic divergence, Proteome, Transcriptome

## Abstract

**Background:**

Lower selection intensities in indigenous breeds of Chinese pig have resulted in obvious genetic and phenotypic divergence. One such breed, the Nanyang black pig, is renowned for its high lipid deposition and high genetic divergence, making it an ideal model in which to investigate lipid position trait mechanisms in pigs. An understanding of lipid deposition in pigs might improve pig meat traits in future breeding and promote the selection progress of pigs through modern molecular breeding techniques. Here, transcriptome and *tandem mass tag-based quantitative proteome* (*TMT)*-based proteome analyses were carried out using *longissimus dorsi* (*LD*) tissues from individual Nanyang black pigs that showed high levels of genetic variation.

**Results:**

A large population of Nanyang black pigs was phenotyped using multi-production trait indexes, and six pigs were selected and divided into relatively high and low lipid deposition groups. The combined transcriptomic and proteomic data identified 15 candidate genes that determine lipid deposition genetic divergence. Among them, *FASN*, *CAT*, and *SLC25A20* were the main causal candidate genes. The other genes could be divided into lipid deposition-related genes (*BDH2, FASN, CAT, DHCR24, ACACA, GK, SQLE, ACSL4,* and *SCD*), PPARA-centered fat metabolism regulatory factors (*PPARA, UCP3*), transcription or translation regulators (*SLC25A20, PDK4, CEBPA*), as well as integrin, structural proteins, and signal transduction-related genes (*EGFR*).

**Conclusions:**

This multi-omics data set has provided a valuable resource for future analysis of lipid deposition traits, which might improve pig meat traits in future breeding and promote the selection progress in pigs, especially in Nanyang black pigs.

**Supplementary Information:**

The online version contains supplementary material available at 10.1186/s12864-021-07764-2.

## Background

In pigs, lipid deposition is a complex and economically important trait that has evolved alongside the fattening efficiency, meat quality, reproductive performance, and immunity traits [1–3]. Subcutaneous, visceral, and intramuscular adipose tissues deposited within muscle fibers, well known as intramuscular fat (IMF or marbling), are the major components of the lipid deposition trait in pigs. Although these lipid tissues have unique metabolic mechanisms [4], they maintain a positive genetic correlation with the subcutaneous, visceral, and intramuscular adipose tissues [5–7]. Current commercial breeds such as Landrace and Yorkshire have undergone long-term and high-intensity selection processes for growth rate and muscle deposition characteristics, and this has resulted in a low lipid deposition trait. An improved understanding of lipid deposition in pigs might improve pig meat quality traits for future breeding and help to improve pig selection when using modern molecular breeding techniques.

A comparative analysis between extreme IMF content phenotypes in Iberian × Landrace crossbred pigs has helped to identify genetic variant locus associated with lipid deposition [8]. Furthermore, three pairs of full-sibling Danish Landrace pigs with extreme opposite backfat thickness phenotypes were also recently compared as well as the prenatal muscle transcriptomes of Tibetan pigs, Wujin pigs, and large White pigs [9, 10]. Xing et al. explored the underlying mechanisms between Songliao black and Landrace pigs using a multi-omics approach, including DNA-seq and RNA-seq [9, 11, 12]. Although several studies have previously attempted to identify genes and pathways involved in lipid deposition traits, to the best of our knowledge, sufficient phenotyping samples are currently lacking or do not consider the noise from the different genetic backgrounds, especially between western commercial and Chinese indigenous breeds.

Compared with Western commercial pigs, Chinese indigenous pigs exhibit a slower growth rate and less lean meat content, but they have superior lipid deposition. Lower selection intensity in Chinese indigenous breeds has resulted in obvious genetic and phenotypic differentiation [11]. The Nanyang black breed of pig is indigenous to the central region of China [13]. Mineral content, marble stripes, meat color, and IMF content in Nanyang black pigs is significantly higher than those in imported breeds (*P* < 0.01) [14–16]. The Nanyang black pig is, thus, an ideal research model for lipid deposition. Considering that obesity poses an escalating health threat worldwide, a deeper understanding of the mechanisms underlying lipid deposition and metabolic changes would be beneficial. To explain the differences in lipid deposition, we identified pairs of Nanyang black pigs with divergent lipid deposition traits and established a lipid genetic differentiation model. Longissimus dorsi (LD) skeletal muscle is one of the largest skeletal muscles of the back spanning the entire thoracic and lumbar regions and has previously been used to evaluate meat quality in the meat processing industry [17, 18]. Transcriptome and proteomic profiling of the *longissimus dorsi* (LD) tissues from Nanyang black pigs with divergent phenotypes was performed to screen candidate genes for lipid deposition. This study focused on the identification of candidate genes that influence lipid deposition and provides crucial expression information for the molecular mechanisms of adipose deposition traits in pigs.

## Results

### Phenotypes of two groups of Nanyang black pigs with divergent lipid depositions

**Fig. 1:**
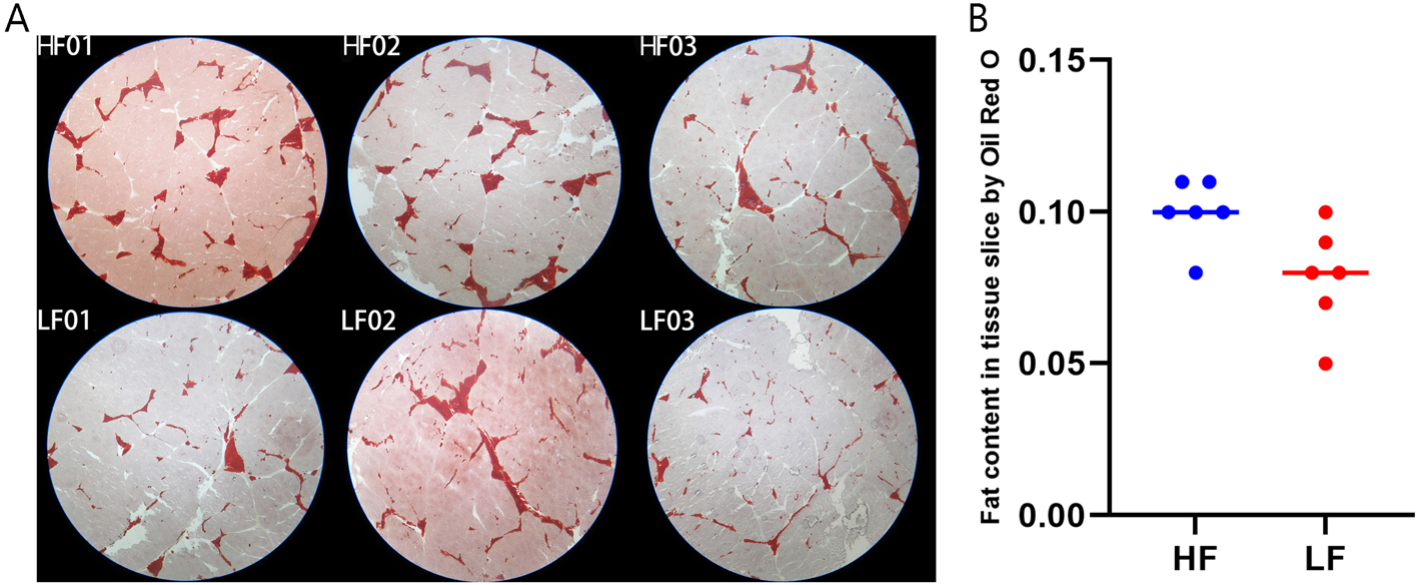
Oil red O staining and fatty acid analysis in *longissimus dorsi* (LD) tissue. **A**: Oil red O staining using frozen LD samples from each of the 6 pigs, HF: high-fat deposition group, LF: low-fat deposition group; **B**: Statistical analysis of the ratio of Oil red O-stained regions using students’ T test. Magnification: 16 ×

Lipid deposition traits in the LD tissue of the Nanyang black pigs with high-and low-lipid-depositions are shown in Table 1 and Fig. 1. Lipid deposition-related traits such as IMF and fat content were determined for the tissue slices using the Soxhlet extraction process and freezing sections and were found to be significantly different between the two groups (*P* < 0.05). The backfat thickness of the live and slaughtered, TFA, and TFA/total dry matter showed the same trend between the high and low lipid deposition groups, although the difference was not significant. It is of note that the significance level of the tissue slice was higher than that from the IMF measurements. By combining the backfat thickness, IMF, fat content in the issue slices, and total fatty acids (TFA)/total dry matter analyses 6 Nanyang black pigs were selected for further analysis and identified as high-fat deposition (HF) and low-fat deposition (LF) groups.

**Table 1:** Phenotypic data for the slaughter and meat quality of the Nanyang black pigs

Name	High lipid deposition group	Low lipid deposition group	***P-***value
**Age (day)**	196	196	1
**Live weight (kg)**	91.120	88.317	0.517
**Backfat thickness of live (mm)**	49.120	38.033	0.074
**Backfat thickness of slaughter (mm)**	37.610	30.433	0.074
**H**_**2**_**O (g/100 g)**	71.997	72.523	0.474
**IMF (%)**	5.370	4.570	0.043
**Fat content in tissue slice by Oil Red O (%)**	10.010	8.070	0.027
**TFA (g/100 g)**	4.170	3.703	0.120
**TFA/Total dry matter (%)**	1.49	1.35	0.179

H2O (g/100 g): percentage of water content in total matter; IMF: intramuscular fat; Fat content in tissue slice by Oil Red O (%): percentage of Oil Red O-stained field in total slice; TFA: total fatty acids; n = 6 in every group

### Transcriptomic analysis between the high and low lipid deposition groups

The cufflinks program identified a total of 342.8 million clean reads and approximately 94.94% of the clean reads were mapped to the *Sus scrofa* genome sequence. In detail, 52.9–60.4 million clean reads were obtained for each sample, and the mapping rates ranged from 94.75 to 95.17%. The clean Q30 base rate varied from 93.96–94.83% (Additional file 1).

By integrating the Fragments Per Kilobase of exon model per Million mapped fragments (FPKM) values to evaluate the gene expression levels, 25,879 genes were identified, and calculated using the FPKM values; of these, 16,597 were detected in all 6 pigs, and they were referred to as positively expressed genes [19]. To determine the accuracy of the grouping, intra- and inter-group correlation analysis was performed for the gene expression of the six pigs, from the perspective of the FPKM values and count numbers, respectively (Additional file 2 A and B). Regardless of the FPKM value or the number of genes, the high lipid deposition group (HF01, HF02, and HF03) was clustered together first and was clearly separated from the low lipid deposition group (LF01, LF02, and LF03).

**Fig. 2:**
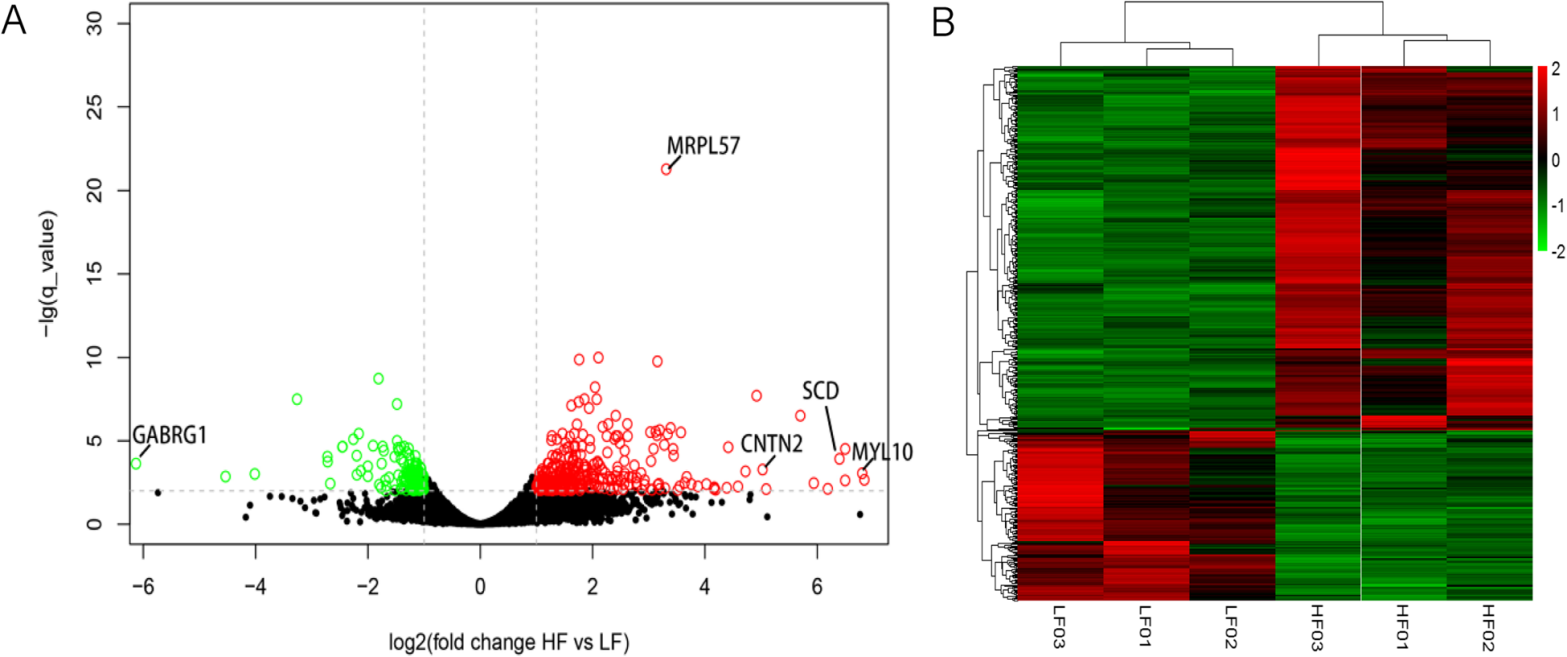
Transcriptome differences between the LD tissue samples from the high and low lipid deposition pigs. **A**: Plot showing the log2 (fold change HF vs. LF) and the –log2 (*q*-value), where the red and green circles indicate the up-and down-regulated DEGs (|log2 fold change| > 1), *q*-value < 0.01); **B**: Heat map of the DEGs in the different lipid deposition groups

There were 481 differentially expressed genes (DEG) identified (|log2 fold change| > 1) that were significant (*q*-value < 0.01). Among them, 331 DEGs had higher expression levels in the HF group than in the LF group, while 150 DEGs displayed opposing tendencies (Fig. 2). Myosin light chain 10 (*MYL10*), Contactin 2 (*CNTN2*), stearoyl-CoA desaturase (*SCD*), and gamma-aminobutyric acid type A receptor gamma1 subunit (*GABRG1)* had large values with |log2 fold changes > 6. *MRPL57* (mitochondrial ribosomal protein L57) was the most significantly differentially expressed gene, with a -log(*q*-value) > 20.

### Functional and clustering annotations of the DEGs

To further utilize the DEG information, they were further interpreted using GO and KEGG analyses to identify the related biological functions and pathways. After integrating the number of clustered genes and the significance levels, skin development, collagen fibril organization, extracellular fibril organization, TBP-class protein binding, and proteasome-activating ATPase activity terms were identified as among the most clustered items (*P* < 0.01) (Additional file 3). KEGG analysis using the DAVID and KOBAS tools helped to validate the 18 most clustered KEGG pathways (gene number ≥ 3, *P* < 0.05) (Additional file 3). Among them were multiple signaling pathways that were involved in lipid formation and metabolism, including fatty acid biosynthesis, PPAR signaling pathway, steroid biosynthesis, fatty acid metabolism, Notch signaling pathway, and the AMPK signaling pathway, which accounted for more than 50% of the significant enrichment pathways. The most significant and maximum number of enriched genes were in the proteasome. The proteasome pathway has important and complex functions, and plays important roles in cell cycle control, apoptosis, oxidative stress, DNA repair, gene transcription regulation, cancer occurrence, and signal transduction. Proteasome degradation has been reported to participate in the relative expression of lipid processing [20, 21]. Overall, the results of the functional analysis revealed that large lipid deposition differences in the two groups, and that the proteasome pathway was the most enriched.

**Fig. 3:**
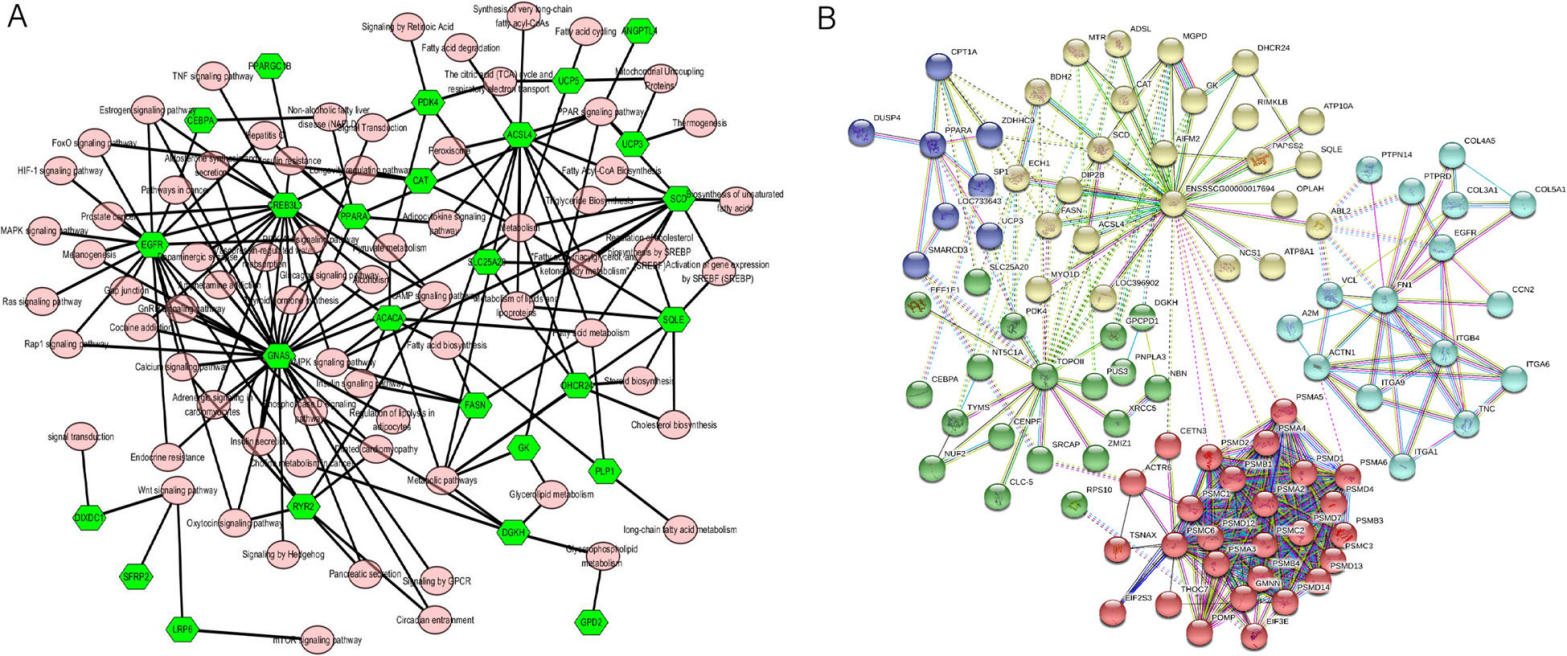
Gene interaction and functional clustering. **A**: Gene interactions with pathways, pink circle: relative pathway, green rhombus; gene symbols; **B**: Gene functional clustering by STRING 11.0, yellow: lipid deposition-related gene; blue: eight PPARA-centered fat metabolism regulatory factors; green: transcription regulators; red: proteolysis-related genes, cyan: integrin genes, structural proteins, and signal transduction-related genes

**Fig. 4:**
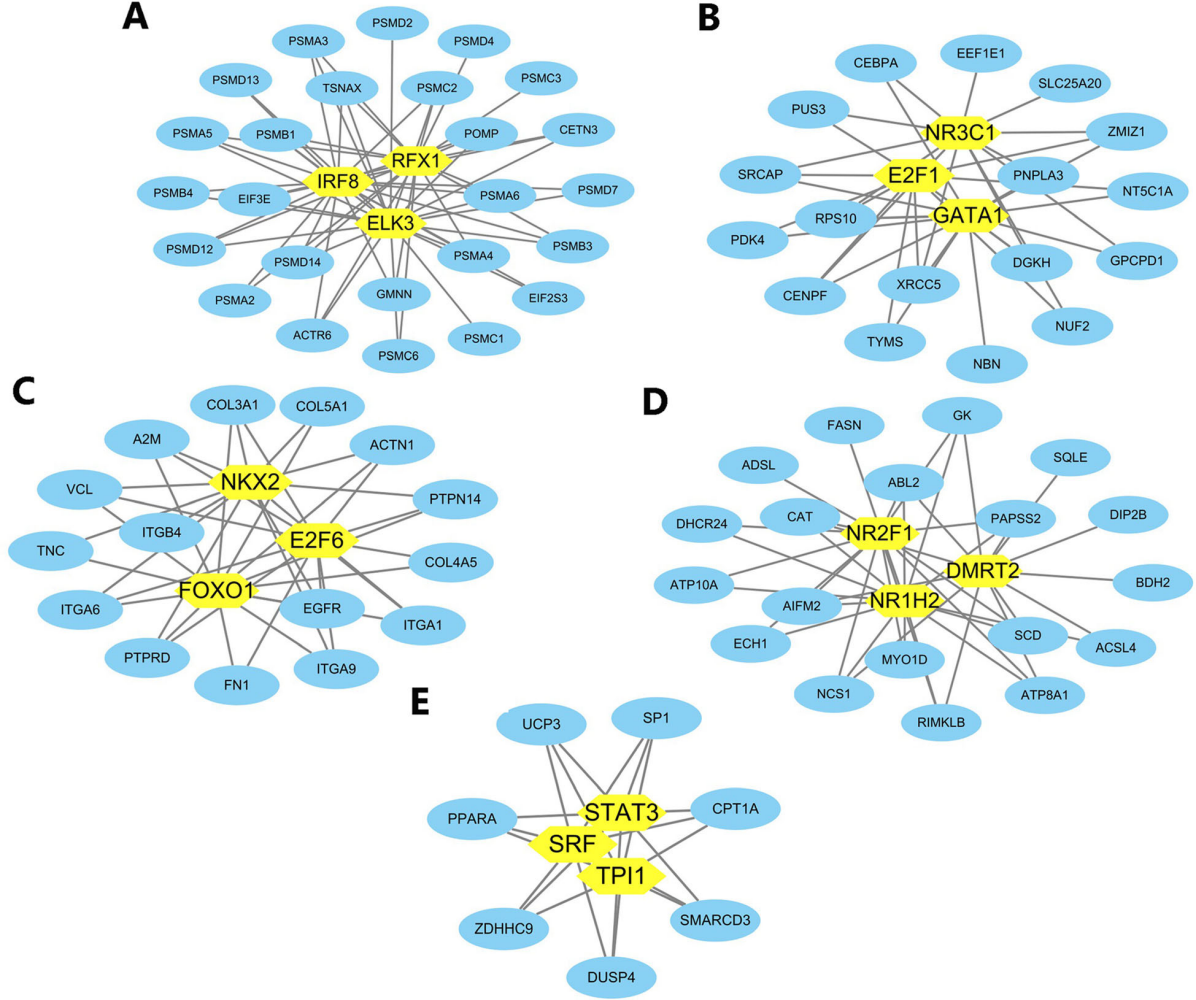
iRegulon analysis of the DEGs from the transcriptomic analysis. All genes analyzed were previously identified in Fig. 3B. Analysis of A: 27 proteolysis-related DEGs; B: 19 transcription regulator-related DEGs; C: 16 integrin genes, structural proteins, and signal transduction-related DEGs; D: 24 lipid deposition-related DEGs; E: 8 PPARA-centered fat metabolism regulatory factor gene-related DEGs

From the KEGG analysis, 26 candidate genes were identified to be involved in the lipid deposition-related pathway, which included peroxisome proliferator activated receptor alpha (*PPARA),* proteolipid protein 1 *(PLP1),* acetyl-CoA carboxylase alpha *(ACACA),* GNAS complex locus *(GNAS),* stearoyl-CoA desaturase *(SCD),* uncoupling protein 3 *(UCP3),* uncoupling protein 5 *(UCP5),* 24-dehydrocholesterol reductase *(DHCR24),* solute carrier family 25 member 20 *(SLC25A20),* pyruvate dehydrogenase kinase 4 *(PDK4),* squalene epoxidase *(SQLE),* secreted frizzled related protein 2 *(SFRP2),* acyl-CoA synthetase long chain family member 4 *(ACSL4),* CCAAT enhancer binding protein alpha *(CEBPA),* glycerol kinase *(GK),* catalase *(CAT),* fatty acid synthase *(FASN),* and epidermal growth factor receptor *(EGFR)* (Fig. 3A, green rhombus). K-means analysis in STRING was also introduced to screen candidate genes. Clustering analysis with *K* = 5 showed that proteolysis-related genes (*red*), transcription regulators (*green*), integrin genes, structural proteins, signal transduction-related gene clusters (*cyan*), lipid deposition-related genes (*yellow*), and the PPARA-centered fat metabolism regulatory factor gene group (*blue*) were enriched (Fig. 3B). All the DEGs from Fig. 3B were used to detect the upstream regulatory TFs and motifs/tracks using iRegulon (Fig. 4). By combining candidate genes from the lipid-related pathways and the K-means analysis in STRING, 14 candidate genes were found to overlap, namely, lipid metabolism genes (*DHCR24, ACACA, GK, CAT, SCD, SQLE, FASN,* and *ACSL*4), transcription regulators (*PDK4, CEBPA*, and *SLC25A20*), PPARA-centered fat metabolism regulatory factors (*PPARA*, *UCP3*), and a signaling transduction gene (*EGFR*).

### Validation of the transcriptome via qRT-PCR

**Fig. 5:**
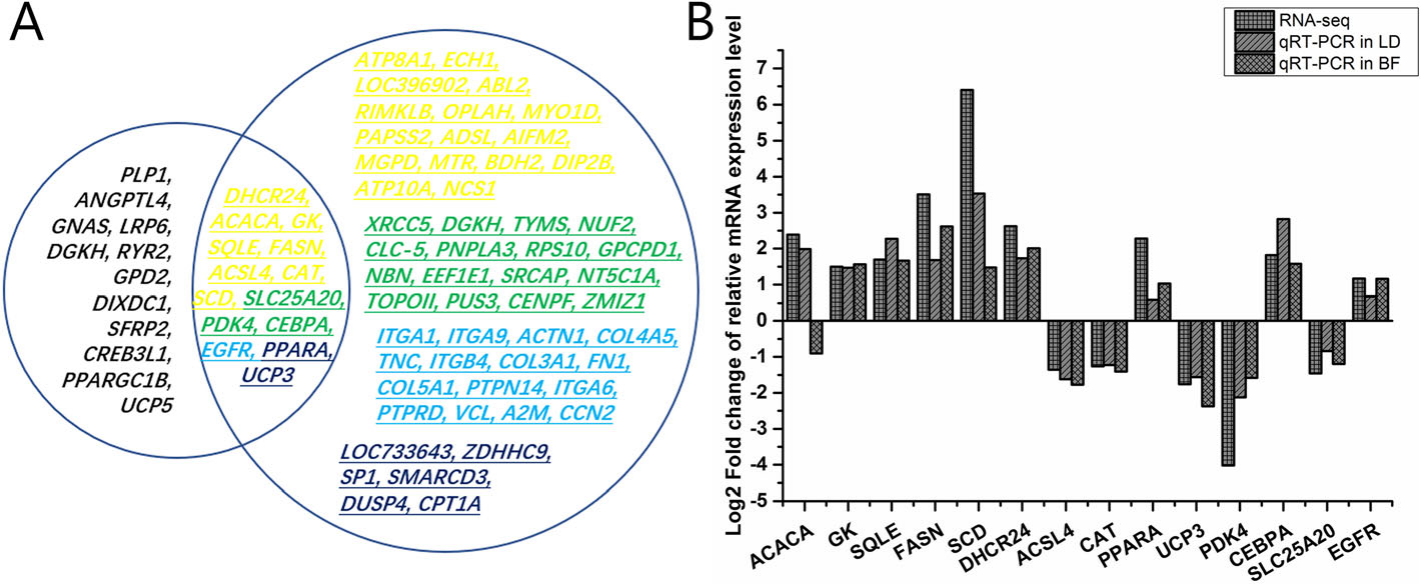
Gene overlapping and validation. **A**: Genes that overlapped between KEGG and STRING. Yellow: lipid deposition-related gene; blue: eight PPARA-centered fat metabolism regulatory factors; green: transcription regulators; cyan: integrin genes, structural proteins, signal transduction-related genes; **B**: qRT-PCR of the 14 DEGs from the LD and backfat (BF) tissues

The expression trends for all 14 genes in the LD tissues were consistent with the results of the transcriptome analysis. In addition to the *ACACA* gene, the expression of the 13 genes from the BF tissue were also consistent with the results of the transcriptome analysis (Fig. 5; Table 2). This showed that the results from the transcriptome sequencing were reliable. And the differences in the expression trends for the *ACACA* gene in the muscle and adipose tissues suggests that it may play a special role in the development of intramuscular fat.

**Table 2:** Log2FoldChanges from the RNA-seq and qRT-PCR analysis of 14 DEGs

	log2FoldChange in RNA-seq	*q* value	log2FoldChange in qRT-PCR of LD	*P* value	log2FoldChange in qRT-PCR of backfat tissue	*P* value
ACACA	2.390	7.6381E-06	1.989	0.021	−0.903	0.044
GK	1.498	0.00504505	1.468	0.040	1.566	0.027
SQLE	1.695	0.00123394	2.271	0.038	1.670	0.040
FASN	3.513	0.00887994	1.678	0.039	2.620	0.049
SCD	6.395	0.00011995	3.529	0.024	1.478	0.038
DHCR24	2.623	5.1834E-06	1.732	0.034	2.011	0.002
ACSL4	−1.360	0.00237211	− 1.623	0.030	− 1.774	0.004
CAT	−1.264	0.00027508	−1.224	0.018	−1.410	0.003
PPARA	2.278	9.7765E-07	0.593	0.034	1.033	0.041
UCP3	−1.756	0.00023243	−1.564	0.040	−2.374	0.010
PDK4	−4.015	0.00096681	−2.125	0.019	−1.580	0.045
CEBPA	1.821	0.004614	2.822	0.042	1.578	0.023
SLC25A20	−1.458	0.00114154	−0.838	0.025	−1.193	0.048
EGFR	1.172	0.00289223	0.677	0.041	1.165	0.020

### TMT-based proteomic analysis between high and low lipid deposition groups

We identified 69,815 peptide-spectrum matches (PSM) that matched 14,317 peptides, of which 11,467 were unique single peptides, and there were 2036 quantified proteins (Additional file 4 A). Most of the proteins were identified by 1–10 peptides (Additional file 4 B). The correlation coefficient is an important parameter when measuring the clusters between samples. As shown in Additional file 4 C, the variation between the biological replicates was small, especially in the high lipid deposition group. Intra-group correlation is an important parameter when measuring reproducibility within a group. The intra-group correlation was higher than the correlation between the groups, and this could be useful for subsequent data analysis.

**Fig. 6:**
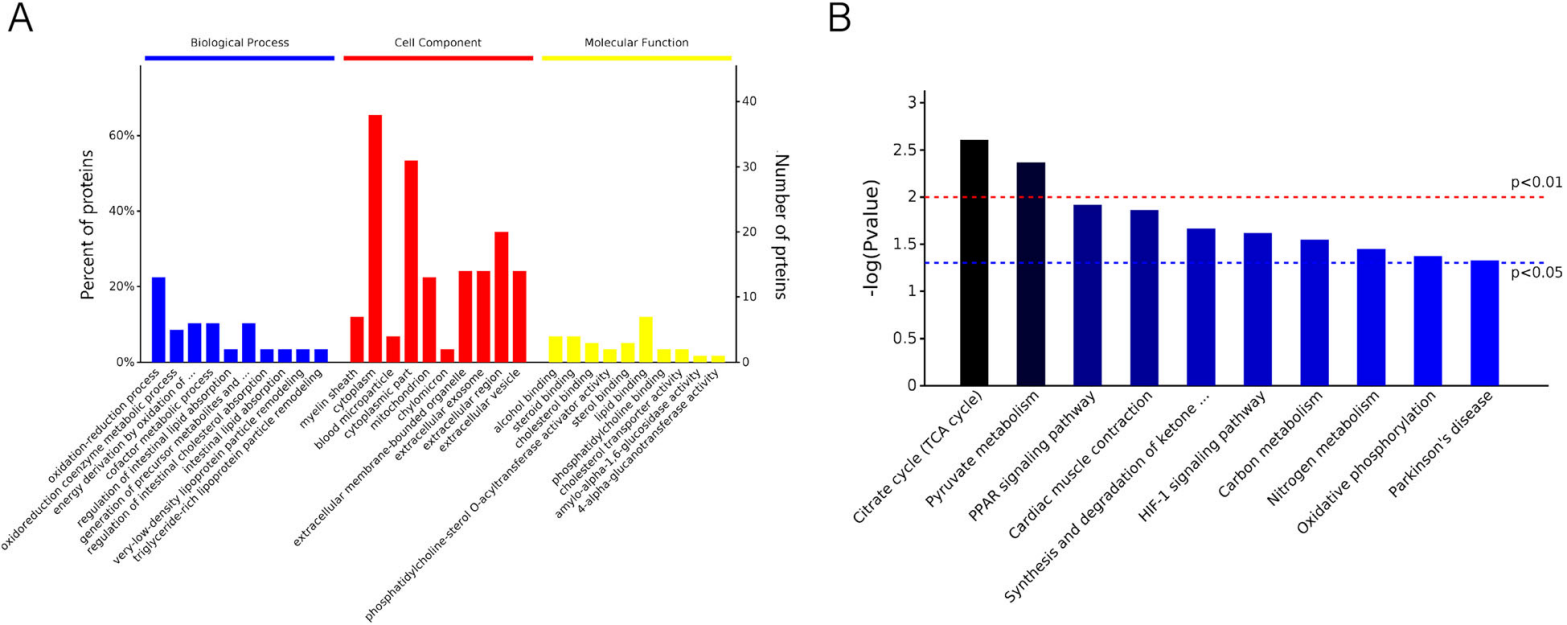
Differentially expressed protein identification and function analysis. **A**: GO analysis of the DEPs. **B**: KEGG analysis of the DEPs

The DEP analysis identified 99 DEPs, of which 63 were upregulated in the HF group and 36 were downregulated (Additional file 5). The 99 DEPs were analyzed using the QuickGO website (Additional file 6). Most were found to be involved in precursor metabolites and energy production, redox reactions, phosphate metabolism processes, phosphorylation, energy production by oxidation of organic components, oxidative phosphorylation, cellular respiration, and electron transport (Fig. 6A). Among them, BP had the most significant enrichment in redox reactions, energy metabolism, and fat absorption and metabolism, while MF had the most significant enrichment in steroid hormone binding and lipid binding. The KEGG functional enrichment analysis of the DEPs revealed that the TCA cycle, pyruvate metabolism, and PPAR signaling pathways, myocardial contraction, ketone body synthesis and metabolism, HIF-1 signaling pathway, carbon and nitrogen cycle, oxidative phosphorylation, and Parkinson’s syndrome (Fig. 6B). Based on the functional analysis of the DEPs, 9 were screened for further analysis, including 3-hydroxybutyrate dehydrogenase 2 *(BDH2)*, *FASN*, *SLC25A20*, eukaryotic translation initiation factor 3 subunit E (*EIF3E*), *CAT*, periaxin (*PRX*), filamin A (*FLNA*), transferrin receptor (*TFRC*), and myelin protein zero (*MPZ*) (Table 3).

**Table 3:** Statistics for the candidate genes identified from the transcriptome and proteome

Gene name	log2FC of mRNA	***q***-value	FC of protein	***P***-value	Annotated pathways
***BDH2***	−1.2750	0.0000	0.6771	0.0425	Synthesis and degradation of ketone bodies, butanoate metabolism, Metabolic pathways
***FASN***	3.5126	0.0089	1.3604	0.0213	Fatty acid biosynthesis, Metabolic pathways, Insulin signaling pathway
***SLC25A20***	−1.4577	0.0011	0.7753	0.0326	Fatty acid oxidation, Metabolism of lipids and lipoproteins, Thermogenesis, Fatty acid, triacylglycerol, and ketone body metabolism, Metabolic pathways,
***EIF3E***	−1.2736	0.0007	1.2355	0.0263	RNA transport, Hepatitis C, mTOR Pathway
***CAT***	−1.2643	0.0003	1.5619	0.0351	FoxO signaling pathway, glyoxylate and dicarboxylate metabolism, Metabolic pathways, Carbon metabolism, Longevity regulating pathway, Amyotrophic lateral sclerosis (ALS)
***PRX***	1.4445	0.0042	1.3672	0.0068	Regulation of RNA splicing
***FLNA***	1.6611	0.0007	1.2641	0.0129	MAPK signaling pathway, Focal adhesion, Salmonella infection, Proteoglycans in cancer, Cytoskeletal Signaling
***TFRC***	1.9311	0.0116	1.9358	0.0218	HIF-1 signaling pathway, Endocytosis, Phagosome, Hematopoietic cell lineage
***MPZ***	3.0469	0.0084	22.725	0.0280	Cell adhesion molecules (CAMs), Neural crest differentiation

log2FC of mRNA: log2FC value between HF and LF group in transcriptome; *q*-value: adjusted *P* value in transcriptome; FC of protein: fold-change value between HF and LF group in proteomic

### Candidate gene screening with the combined transcriptome and proteome data

**Fig. 7:**
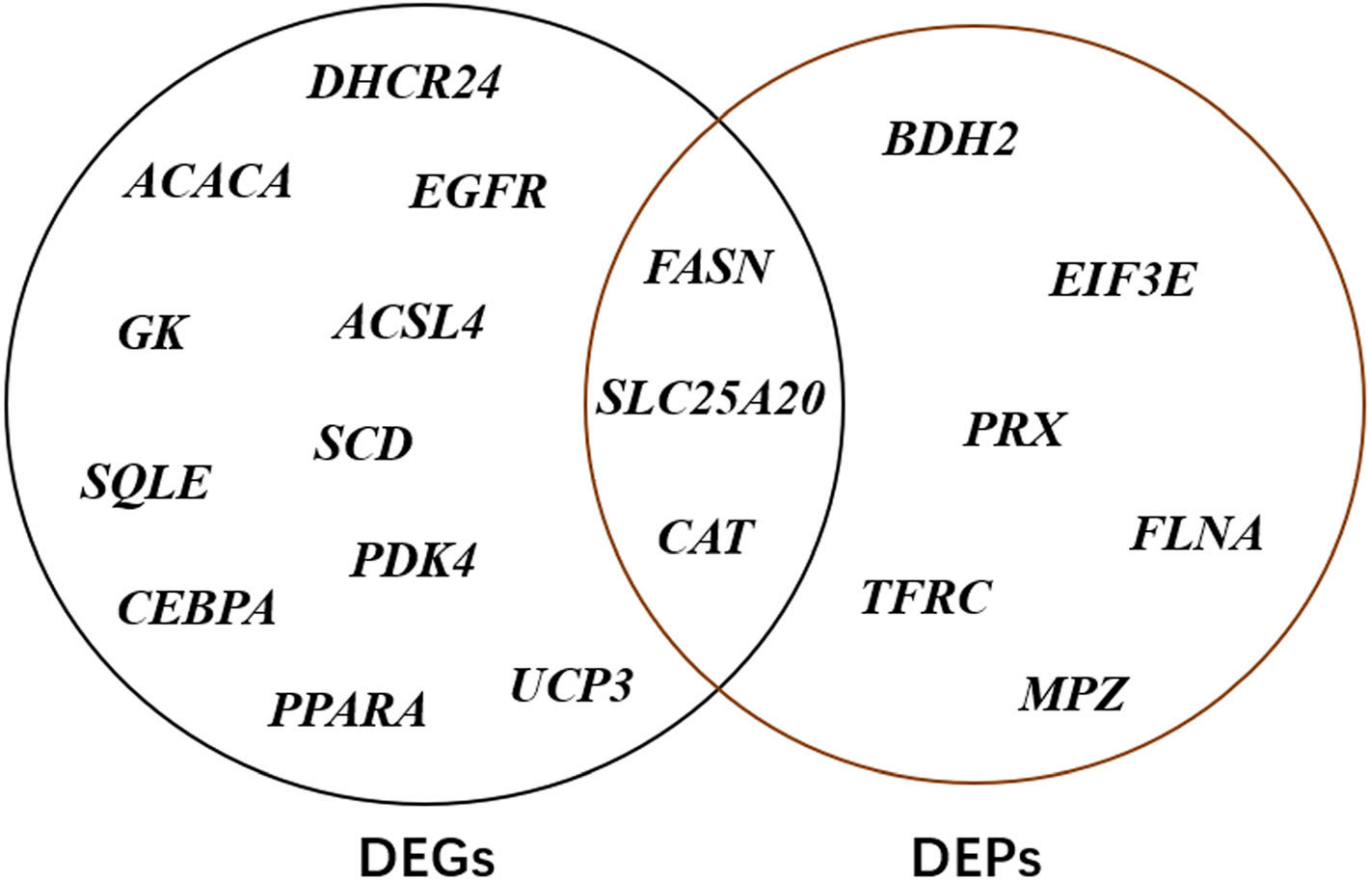
Venn plot of the candidate proteins and DEGs for lipid deposition

A Venn diagram was produced for the lipid deposition-related candidate DEPs and DEGs, and it showed that three genes overlapped, *FASN, CAT,* and *SLC25A20*, and they were identified as lipid deposition related genes (Fig. 7). While FASN and CAT displayed a consistent tendency between the mRNA and protein, *SLC25A20* displayed the opposite tendency. Moreover, several DEGs were not detected in the proteomic analysis, including *DHCR24, ACACA, GK*, and *UCP3.*

## Discussion

Asian wild pigs were derived from ancient wild boars approximately 1.2–0.8 million years ago and the domestication of the pig in China occurred ∼9000 years ago [22, 23]. Nanyang black pigs are one of the three main Chinese indigenous pig breeds in Henan Province and the quality of their meat is higher than that of Western commercial breeds (China National Commission of Animal Genetic Resources 2011) [15]. Lower selection intensity in Nanyang black pigs has resulted in obvious genetic and phenotypic differentiation, especially in lipid deposition traits [11]. Genetic diversity provides the basic information required for research into genetics and breeding [17, 24, 25]. Consequently, the detailed genetic mechanisms for lipid deposition in Nanyang black pigs requires further investigation. In the present study, live screening was performed, and phenotypic differences between the groups were confirmed by assessing their slaughtering backfat thickness, IMF, fatty acid, and Oil Red O staining. The results showed that the Nanyang black pigs were suitable to screen for genes related to fat deposition. Subsequently, a multiple omics method was adopted to compare high and low lipid deposition groups sourced from a local pig breeding farm. Although there have been several comparative studies on pig lipid deposition and several lipid-related genes have been reported, most of these investigations have been carried by analyzing the differences between species or between different physiological stages [3, 10, 17, 24–28].

According to the central dogma of molecular biology, the process from genome to transcriptome to proteome is a step-by-step process that is extremely complex and has been perfected throughout evolution. The transcriptome is sensitive and can identify almost all genes expressed in the tissue. Here, the number of positively expressed genes identified in this study was 16,579; however, coding genes need to be translated into proteins, so further screening using proteomics was required. Transcriptome responses may be triggered by a variety of factors, such as transcription factors [12]. While the current sensitivity of proteomics is not currently precise enough to detect all proteins. After database comparisons, the number of positively expressed proteins identified was 2036, which was lower than that of the transcriptome. Although some DEPs (*CMYA1, IGHG, LOC100522678, and LOC100623720*) were not detected in transcript level, all of them were mot well annotated (https://www.ncbi.nlm.nih.gov/) [29]. Theoretically, despite complex post-transcriptional controls, the relationship between mRNA and protein levels should be positively correlated. Previous studies have reported a medium or low positive correlation between the transcriptome and proteome [10, 30, 31]. In this study, the correlation index between DEGs from the RNA-seq and DEPs from the TMT analysis was approximately 0.7 (*P* < 0.05), which indicates that the high-throughput data was of high quality and the screening results were moderately reliable. While the combined analysis of the transcriptome and proteome data can provide more accurate and comprehensive gene expression information than single omics data, some genes from the single omics results were also discussed here to compliment the combined results [12, 19]. Here, we have focused on screening the candidate genes regulating lipid deposition by combining DEGs and DEPs.

In this investigation, the DEGs and DEPs of *FASN*, *SLC25A20*, and *CAT* were found to overlap. *FASN*, encoded by a gene located in a QTL region associated with fatty acid composition and involved in fatty acid metabolism, has been widely reported as a marker gene for lipogenesis in cattle [32], mice [33], rats [34], and pigs [35]. *FASN* was expressed at significantly higher levels in the high fatty acid group, which was in accordance with its fatty-accumulating functions that were identified in previous studies [35, 36]. Consistent expression tendencies for the *FASN* also helped to validate the accuracy of our multi-omics studies. *CAT*, a key regulator of oxidative stress, also showed higher levels in the high lipid deposition group. This indicates that it is upregulated to compensate for the H_2_O_2_ accumulation induced by the high lipid levels. Generally, the oxidative status of pork helps to determine its pH value after slaughter, drop loss, and IMF content [37]. Additionally, endogenous catalase regulates the polarization of adipose macrophages, thus inhibiting inflammation and insulin resistance in humans [38]. Catalase-knockout mice showed exacerbated insulin resistance, amplified oxidative stress, and accelerated macrophage infiltration into white adipose tissues [38]. Unlike *FASN* and *SLC25A20*, *CAT* displayed a divergent expression pattern between its mRNAs and proteins, indicating complex post-transcriptome regulatory mechanisms and functional networks either from multiple-omics [39] or *CAT* analysis [40]. PPAR signaling pathways were significantly clustered with *P* < 0.05 in the DEPs covering *FASN* and *SLC25A20*. *SLC25A20* is a key molecule that transfers acyl-carnitine esters to free carnitine across the mitochondrial membrane during mitochondrial beta-oxidation. *SLC25A20*, like *FABP4*, *ACOX1*, *CYP4A24*, and *PDK4*, is also known as a *PPARA* target gene, which had a main function of fatty acid β-oxidation [41]. In all, *FASN*, *CAT,* and *SLC25A20* were all causal genes determining lipid deposition in LD.

*BDH2*, was also identified as a DEG and DEP; however, functional analysis of the DEGs involved in lipid deposition missed *BDH2*. *BDH2* is distinct from mitochondrial type-*BDH1*, as it plays a role in cytosolic ketone body utilization and in secondary systems for energy supply during starvation [42]. For lipid deposition, *BDH2* expression was reported to be positively associated with adiposity by generating precursors for lipid and sterol synthesis [43, 44]. However, *BDH2* is also regarded as a fatty acid oxidation gene functioning with *CYP4A3* [45]. In the present study, the mRNA and protein levels of BDH2 were all significantly downregulated in the high lipid deposition group, suggesting that *BDH2* in the Nanyang black pigs was more likely to be related to fatty acid oxidation and could be identified as a candidate gene for lipid traits. The detailed mechanisms for these processes require further analysis. Upstream transcriptional factor analysis of the 24-lipid deposition-related genes showed that *NR2F1*, *NR1H2*, and *DMRT2* were mainly clustered (NES > 5.042) by *FASN, SCD, ACSL4, CAT,* and *BDH2*. *NR2F1* and *NR1H2* are best known as nuclear oxysterol receptors and physiological master regulators of lipid and cholesterol metabolism [46], while DMRT2 was mainly reported as a myogenic regulator [47]. Here, we screened three upstream transcriptional factors regulating lipid deposition-related DEGs, especially DMRT2.

Other DEGs (*DHCR24*, *ACACA*, *GK*, *SQLE*, *ACSL4*, *SCD*, *PDK4*, *CEBPA*, *EGFR*, *PPARA*, and *UCP3*) were screened using KEGG and K-means algorithms. Unfortunately, the TMT-based quantitative proteomics could not detect them. Most of them showed overlaps with previous omics studies, especially *ACACA*, *ACSL4*, *SCD*, *PDK4*, *CEBPA*, *EGFR*, *PPARA*, and *UCP3* [10, 26, 48, 49]. *DHCR24* and *SQLE* are involved in lipid metabolism and cholesterol synthesis, as reported previously for granulosa cells through FSH and FOXO1 [50]. The upregulation of *GK* is related to higher lipid biosynthesis [51, 52]. In the present study, all the genes mentioned were upregulated in the HF group, in addition to *ACSL4*, *UCP3*, and *PDK4*, which had also been reported in previous investigations [32, 53]. As reported previously, *ACSL4* and *UCP3* were associated with lipogenesis. It should be noted that *ACACA* displayed an opposite tendency in the LD and backfat tissues and high expression levels in the HF groups LD tissue and the LF groups backfat tissue; a similar phenomenon was observed in a previous investigation [54]. ACACA was also a key lipogenic enzyme involved in hepatic lipid deposition. There have been many controversial studies of the multiple roles involving ACACA in mono- and poly-unsaturated fatty acid content and performance traits [54, 55]. Stachowiak et al. further reported that ACACA shows a distinct expression pattern in the subcutaneous fat and LD muscle of Landrace pigs [56]. This indicates that *ACACA* might be involved in determining the directional deposition of the lipids and this should be investigated further in the future.

## Conclusions

In conclusion, we identified 481 DEGs using high-quality RNA-seq and 99 DEPs using a TMT-based quantitative proteomic analysis. By combining the transcriptome and proteome profiles, 15 genes were identified as being associated with genetic divergence. These genes were divided into lipid deposition-related genes (*BDH2*, *FASN*, *CAT*, *DHCR24*, *ACACA*, *GK*, *SQLE*, *ACSL4*, and *SCD*), PPARA-centered fat metabolism regulatory factors (*PPARA* and *UCP3*), transcription or translation regulators (*SLC25A20*, *PDK4*, and *CEBPA*), integrin, structural proteins, and signal transduction-related genes (*EGFR*). Among them, *FASN*, *CAT*, and *SLC25A20* were the main causal candidate genes. Upstream transcriptional factor analysis validated the three-lipid deposition-related genes *NR2F1*, *NR1H2*, and *DMRT2.* According to the results obtained in the present study, the genetic mechanisms of divergence in the Nanyang black pigs are complex and determined by multiple genes. This study provides valuable information for further research of the molecular mechanisms underlying porcine lipid deposition traits, especially those for Nanyang black pigs. Taking advantage of the causal genes for lipid deposition could improve the breeding of Nanyang black pigs and help to preserve Chinese indigenous breeds.

## Materials and methods

### Ethics statement

The experimental 12 pigs used were all obtained from a national elite reservation farm in Neixiang, MuYuan Foods co ltd, China, as per their permission. Slaughter and sampling were all carried out under tight supervision to minimize animal suffering. The Animal Welfare Committee of the State Key Laboratory for Agro-Biotechnology of the China Agricultural University approved all procedures for animal care (approval number, SKLAB-2012-04-07). Furthermore, all experiments were conducted in accordance with approved relevant guidelines and regulations during slaughter, sampling, and sample conservation.

### Animals

The Nanyang black pig population composing of 12 Nanyang black pigs was in Neixiang county of Nanyang City and were all housed for their lifespan in the standard environmental conditions, with a natural, uncontrolled room temperature. All diets were formulated to provide essential nutrients to meet NRC requirements of China in 2012. The relative humidity and temperature of the piglet houses were maintained at 60–65% and 25–28 °C, respectively. The animals were fed three times a day and had access to water ad libitum. Pedigree information is available for all animals. Backfat thickness between the 3rd and 4th last ribs of sibling female pigs from a pen was measured using real-time B-mode ultrasonography with an HS1500 convex scanner (Honda Electronics, Toyohashi, Japan). All the twelve pigs were slaughtered and the LD and backfat tissues were excised and sampled for qRT-PCR. Six pigs were used for IMF measurements, cryotome observations, and transcriptome and proteome analysis. Backfat thickness was measured using Vernier calipers.

### Phenotype measurements and histological observations

To evaluate the production performance of the sows, especially their lipid deposition traits, we measured IMF using the Soxhlet extraction method, as previously described [18, 19]. Their fatty acids were also measured using gas chromatography-mass spectrometry (GC-MS) (Agilent 7890A, CA, USA). Measurements of the 6 individuals were performed using three technical replicates. Samples of LD muscle stored at − 80 °C were embedded in optimum cutting temperature (OCT) compound and dissected along the horizontal axis into 19–20 nm thick pieces. The frozen sections were then stained with Oil Red O and hematoxylin eosin for 5 min and 1 min, respectively [18]. Viewing and imaging were conducted using a microscope (× 1.6; Nikon, Tokyo, Japan) in a white field; three fields of horizon were selected randomly and saved for later statistical analysis using Image J (Version:1.8.0).

### RNA extraction and sequencing

LD and backfat samples were homogenized, and RNAs were extracted in Trizol (Invitrogen, USA) according to the manufacturer’s instructions. Isolated total RNA was quantified (Nanodrop, ND2000) and quality controlled with typical curves (Agilent, Bioanalyzer 2100). Only high-quality RNA (RNA integrity number, RIN > 7.0) was used to construct the cDNA libraries (TruSeq RNA Sample Preparation Guide, Illumina Inc., San Diego, CA). All libraries were sequenced on a HiSeq 4000 (Illumina Inc., San Diego, CA, USA) with PE (paired end; 150 bp). The obtained raw data were filtered to clean data with fastp (version 0.12.3) by removing reads containing adapters, low-quality reads, and reads containing more than 5% N (default parameters), and they were then mapped to the pig reference genome of *Sus scrofa 11.1.92* (ftp://ftp.ensembl.org/pub/release-92/fasta/sus_scrofa/dna/Sus_scrofa.Sscrofa11.1.dna.toplevel.fa.gz) using HISAT2 (version 2.0.5) [57, 58]. The number of fragments per kilobase of the transcripts per million mapped reads (FPKM) was used to determine the levels of gene expression with cufflinks (version 2.2.1) [59]. The HTSeq (version 0.6.1) [60] using “intersection-strict” mode and a minimum alignment quality of 10 was used to construct the read counts matrix as the DESeq2 input data. DESeq2 [61] with outlier replacements and independent filtering was adopted to detect the differentially expressed transcripts between the high and low lipid deposition groups. The differentially expressed genes (DEGs) between the groups were identified using a statistical significance of |log2 fold change| > 1 and *q*-value (adjustment for *P* value) < 0.01.

### Functional annotation of differentially expressed genes

Gene Ontology (GO) terms and the Kyoto Encyclopedia of Genes and Genomes (KEGG) pathways were used to annotate the transcriptome results. DAVID 6.8 [62] and KOBAS3.0 [63] were adopted, and only GO terms or KEGG pathways that were found to overlap by the two websites were regarded as candidates (*P* < 0.05). Moreover, interaction networks between the DEGs were also analyzed using STRING11.0 [64]. Clustering of the STRING networks was performed using an embedded k-means algorithm, with a number of expected clusters determined empirically [65]. Furthermore, to screen the possible regulators or transcription factors that were activating or inhibiting gene expressing, iRegulon v1.3. was used to identify transcription factors regulating DEGs expressing in silico [66]. Gene interaction diagrams were constructed using Cytoscape 3.8.2 (http://www.cytoscape.org/) according to the manufacturer’s instructions.

### Quantitative real-time PCR (qRT-PCR)

Total RNA was extracted for qRT-PCR analysis in the LD and backfat tissues of the 12 pigs, which included the high lipid group (*n* = 6) and the low lipid group (n = 6). In all, 14 DEGs were chosen randomly for qRT-PCR (*ACACA, GK, SQLE, FASN, SCD, DHCR24, ACSL4, CAT, PPARA, UCP3, PDK4, CEBPA, SLC25A20,* and *EGFR*) (Additional file 7). Among them, *ACACA, GK, SQLE, FASN, SCD, DHCR24, PPARA, CEBPA,* and *EGFR* were upregulated, while *ACSL4, CAT, USP3, PDK4,* and *SLC25A20* were downregulated in the high-lipid group. All experiments were performed in triplicates. Relative gene expression levels were normalized to the levels of GAPDH and HPRT. The 2^−ΔΔCt^ method was used to evaluate the relative gene expression levels [67]. All experiments were carried out using the CFX96TM Real-Time System (Bio-Rad, Hercules, CA, USA). Data are presented as means ± standard error. The differences in the values were evaluated using Duncan’s multiple comparison with a Bonferroni justification by using SAS 9.2. The differences were considered significant at *P* < 0.05 and highly significant at < 0.01.

### Tandem mass tag based proteomics

Isobaric tandem mass tags (TMTs) were used to detect differentially expressed proteins (DEPs) in the pigs with divergent lipid traits [68]. The LD tissue was digested and labeled with TMT labels and then analyzed using liquid chromatography-tandem mass spectrometry (LC-MS/MS). A six-plex TMT strategy and high-performance liquid chromatography (HPLC) fractionation of 15 times followed by LC-MS /MS using Q-Exactive HF-X (Thermo Scientific, CA) was used to identify the DEPs. This process allowed for three simultaneous replicates in a single run, guaranteeing high confidence and robust statistics for quantitative measurements [69].

The LC-MS/MS data were searched for in the “Uniprot_*Sus scrofa*_ 50068–20180925 _Uniprot.fasta” database (https://www.uniprot.org/uniprot/?query=taxonomy:9823) using Max Quant 1.6.0.16 (Thermo Fisher Scientific) for peptide identification and quantification. At least two unique peptides for a unique protein, with a *q-value* < 0.01, were screened for further quantification. The quantification level for the unique peptide was corrected as the proportion of the total intensity of the assigned peptides. Peptide-spectrum match (PSM) filtering was performed using linear discriminant analysis, as described previously [70]. Relative protein expression levels were normalized to the median average peptide ratio. Fold changes of > 1.2, < 0.833, and a *P* < 0.05, were set as the thresholds with which to identify the DEPs. Fisher’s exact test in QuickGO (http://www.ebi.ac.uk/QuickGO/) was used to evaluate the significance level of the GO TERM after the DEP enrichment. KEGG enrichment was carried out using KEGG API (https://www.kegg.jp/kegg/rest/keggapi.html).

### Statistical analysis

The results shown in the tables and figures represent at least three independent trials or until reproducible results were obtained. Data are presented as the mean ± standard error (SE). Student’s t-tests were performed using Statistical Analysis System software (SAS, version 9.2, SAS Institute Inc., Cary NC, USA) to examine the significance of the differential expression levels among the groups, and the differences among the groups were considered significant at *P* < 0.05 and highly significant at *P* < 0.01.

## Supplementary Information

**Additional file 1: Table.** Transcriptome data from *longissimus dorsi* (LD) samples from Nanyang Black pigs. Basic transcriptome summary data for six samples including the Raw reads, Clean reads rate, and mapping rates.

**Additional file 2: Figure.** Correlation analysis of gene expression both between and in groups. FPKM based analysis and count number-based analysis results in transcriptome analysis.

**Additional file 3: Figure.** Functional analysis of the 481 DEGs using GO and KEGG. Results of the GO and KEGG analysis of the 481 DEGs from transcriptome with |log2 fold change| > 1 and *q*-value < 0.01.

**Additional file 4: Figure.** Analysis of TMT-based proteomic analysis. Detailed results from the TMT-based proteomic analysis including the basic information, peptide number, and correlation analysis.

**Additional file 5: Table.** TMT_based proteomic expression analysis of the Nanyang black pigs. Expression analysis of the differentially expressed proteins identified in this investigation.

**Additional file 6: Figure.** Differentially expressed protein identification and functional analysis. Analysis of the 99 differentially expressed proteins identified in this investigation.

**Additional file 7: Table.** Primers used for the qRT-PCR analysis of the DEGs. A list of the primers used to assess the differentially expressed genes composing of ACACA, GK, SQLE, FASN, SCD, DHCR24, ACSL4, CAT, PPARA, UCP3, PDK4, CEBPA, SLC25A20, and EGFR.

## Data Availability

The datasets generated and analyzed during the current study are available in the GEO repository with the accession number: GSE165613 (https://www.ncbi.nlm.nih.gov/geo/query/acc.cgi?acc=GSE165613).
