# Supplementary material for: Candidate gene screening for lipid deposition using combined transcriptomic and proteomic data from Nanyang black pigs
Source: BMC Genomics. 2021 Jun 12;22:441. doi: 10.1186/s12864-021-07764-2 (PMC8201413; doi:10.1186/s12864-021-07764-2)
Supplement: Supplementary file 1 — Additional file 1: Table. Transcriptome data from longissimus dorsi (LD) samples from Nanyang Black pigs. Basic transcriptome summary data for six samples including the Raw reads, Clean reads rate, and mapping rates. [file 12864_2021_7764_MOESM1_ESM.docx]

Additional File 1: Table. Transcriptome data from *longissimus dorsi* (LD) samples in Nanyang Black pigs

| **Sample** | **HF01** | **HF02** | **HF03** | **LF01** | **LF02** | **LF03** |
| --- | --- | --- | --- | --- | --- | --- |
| **Raw reads** | 55,563,730 | 62,048,786 | 59,307,244 | 63,476,448 | 62,333,082 | 58,662,066 |
| **Raw bases** | 8,334,559,500 | 9,307,317,900 | 8,896,086,600 | 9,521,467,200 | 9,349,962,300 | 8,799,309,900 |
| **Clean reads** | 52,920,492 | 58,711,272 | 54,788,332 | 60,436,906 | 59,761,944 | 56,164,520 |
| **Clean reads rate** | 0.95 | 0.94 | 0.92 | 0.95 | 0.95 | 0.95 |
| **Clean bases** | 7,938,073,800 | 8,806,690,800 | 8,218,249,800 | 9,065,535,900 | 8,964,291,600 | 8,424,678,000 |
| **Low quality reads** | 463,834 | 595,480 | 463,882 | 870,012 | 539,086 | 541,134 |
| **Low quality reads rate** | 0.83 | 0.96 | 0.78 | 1.37 | 0.86 | 0.92 |
| **Ns reads n** | 10,030 | 10,956 | 9,900 | 11,520 | 9,876 | 8,862 |
| **Ns reads rate** | 0.02 | 0.02 | 0.02 | 0.02 | 0.02 | 0.01 |
| **Adapter polluted reads** | 2,169,374 | 2,731,078 | 4,045,130 | 2,158,010 | 2,022,176 | 1,947,550 |
| **Raw Q30 bases rate** | 94.15 | 94.3 | 94.54 | 93.3 | 94.01 | 94.01 |
| **Clean Q30 bases rate** | 94.53 | 94.75 | 94.83 | 93.96 | 94.41 | 94.44 |
| **Mapping rates** | 95.07 | 95.02 | 94.81 | 94.75 | 94.84 | 95.17 |

HF: high fat deposition group; LF: low fat deposition group.
