# Supplementary figures and images for: Candidate gene screening for lipid deposition using combined transcriptomic and proteomic data from Nanyang black pigs
Source: BMC Genomics. 2021 Jun 12;22:441. doi: 10.1186/s12864-021-07764-2 (PMC8201413; doi:10.1186/s12864-021-07764-2)

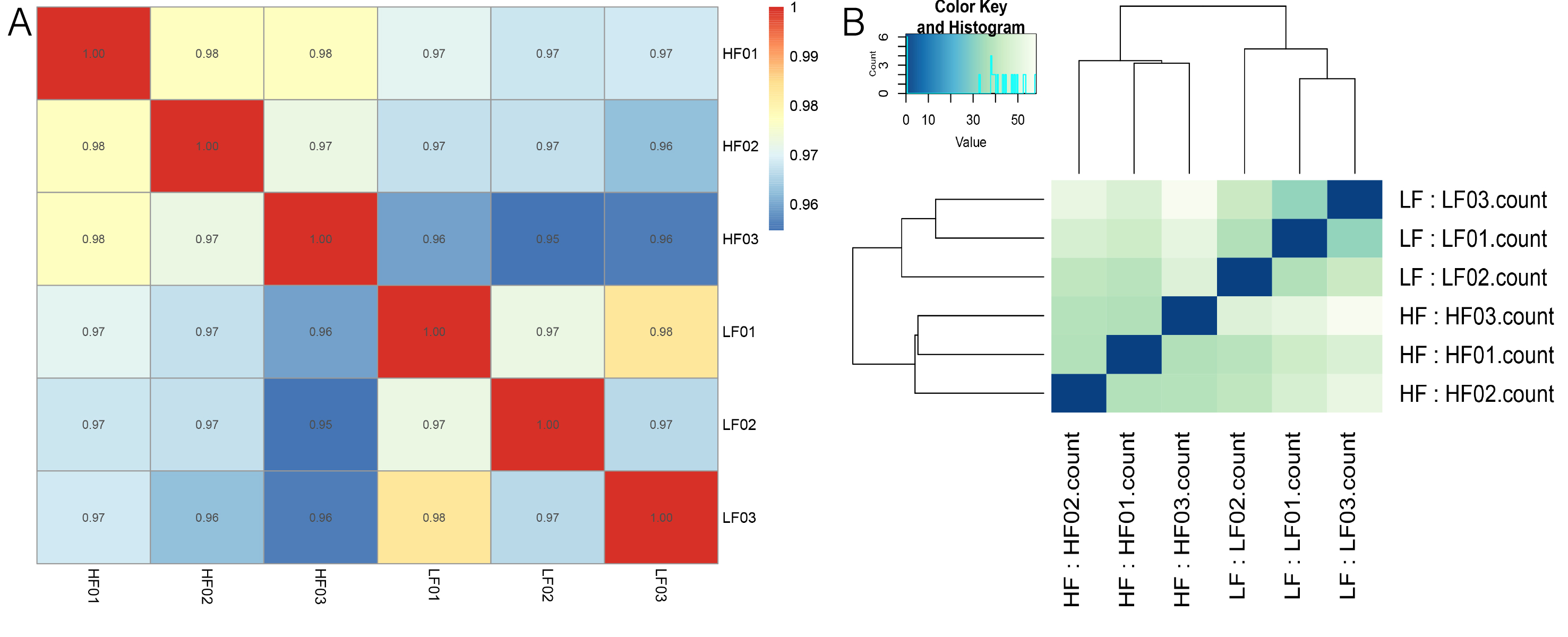

Supplement: Supplementary file 2 — Additional file 2: Figure. Correlation analysis of gene expression both between and in groups. FPKM based analysis and count number-based analysis results in transcriptome analysis. [file 12864_2021_7764_MOESM2_ESM.jpg]

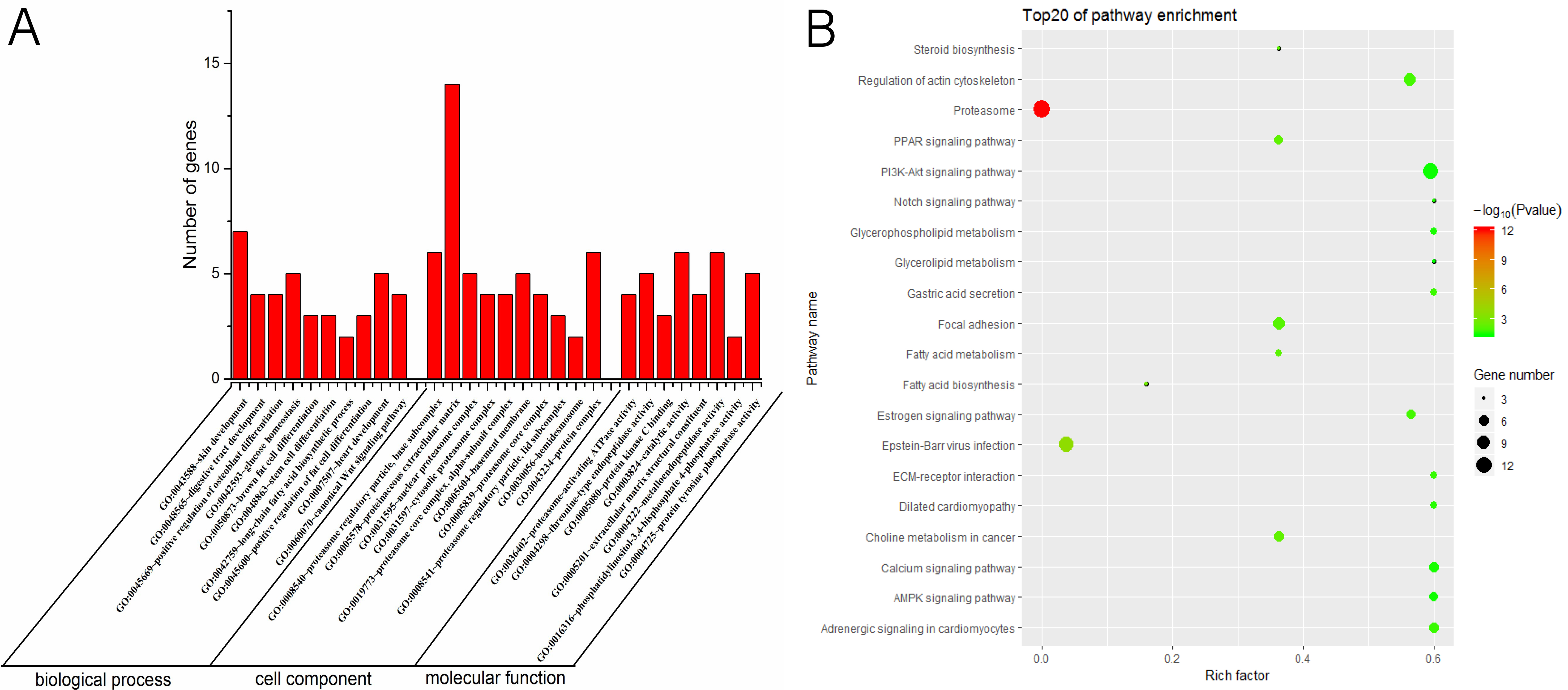

Supplement: Supplementary file 3 — Additional file 3: Figure. Functional analysis of the 481 DEGs using GO and KEGG. Results of the GO and KEGG analysis of the 481 DEGs from transcriptome with |log2 fold change| > 1 and q-value < 0.01. [file 12864_2021_7764_MOESM3_ESM.jpg]

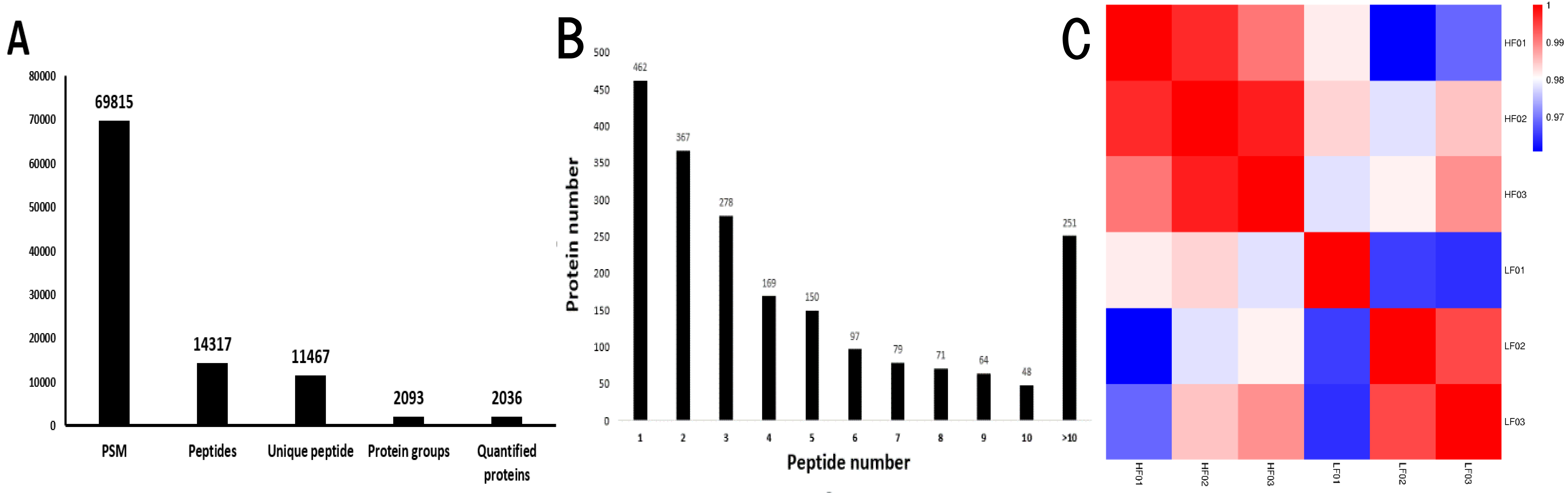

Supplement: Supplementary file 4 — Additional file 4: Figure. Analysis of TMT-based proteomic analysis. Detailed results from the TMT-based proteomic analysis including the basic information, peptide number, and correlation analysis. [file 12864_2021_7764_MOESM4_ESM.jpg]

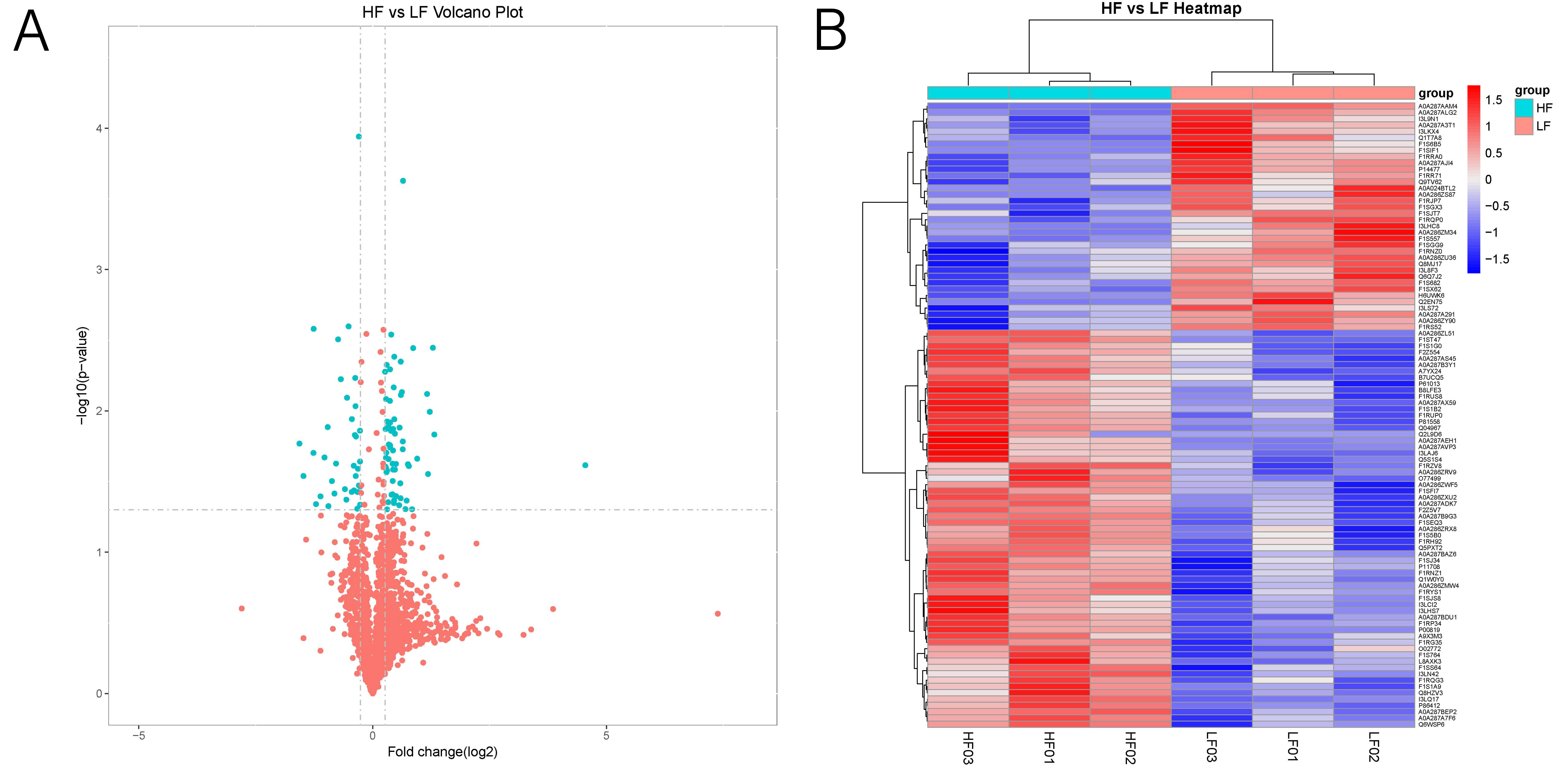

Supplement: Supplementary file 6 — Additional file 6: Figure. Differentially expressed protein identification and functional analysis. Analysis of the 99 differentially expressed proteins identified in this investigation. [file 12864_2021_7764_MOESM6_ESM.jpg]
