# Supplementary material for: Candidate gene screening for lipid deposition using combined transcriptomic and proteomic data from Nanyang black pigs
Source: BMC Genomics. 2021 Jun 12;22:441. doi: 10.1186/s12864-021-07764-2 (PMC8201413; doi:10.1186/s12864-021-07764-2)
Supplement: Supplementary file 7 — Additional file 7: Table. Primers used for the qRT-PCR analysis of the DEGs. A list of the primers used to assess the differentially expressed genes composing of ACACA, GK, SQLE, FASN, SCD, DHCR24, ACSL4, CAT, PPARA, UCP3, PDK4, CEBPA, SLC25A20, and EGFR. [file 12864_2021_7764_MOESM7_ESM.docx]

Additional file 6: Table. Primers used for the qRT-PCR of the DEGs

| Gene symbol | log2Fold-Change | *q-value-*value | Primer Sequence  3’ to 5’ | Length（bp） |
| --- | --- | --- | --- | --- |
| *ACACA* | 2.38994 | 7.64E-06 | TGGAGGAGAAGGAGGG | 126 |
|  |  |  | GGTGCAAGCCAGACAT |  |
| *GK* | 1.497917 | 0.005045 | TAAAGCAGTTTTGGGACC | 105 |
|  |  |  | TGATGATGACTAAGTAGTTCAGC |  |
| *SQLE* | 1.695407 | 0.001234 | CCAGTTCGCCGTCTT | 155 |
|  |  |  | CTGCTCCTACTAAGGTTGTT |  |
| *FASN* | 3.512594 | 0.00888 | CTCCAAGCAGGCGAACACG | 102 |
|  |  |  | CCACGAAGGGAAGCAGGGT |  |
| *SCD* | 6.395106 | 0.00012 | GAGTCACCGAACTTACAAA | 192 |
|  |  |  | AAGCAGCCAACCCAC |  |
| *DHCR24* | 2.623483 | 5.18E-06 | GTGCCTCTTCCTCCTGC | 149 |
|  |  |  | CCTGCTCCTTCCATTCC |  |
| *ACSL4* | -1.3599 | 0.002372 | AAGCACGAACAATAGACA | 123 |
|  |  |  | GACAGAGCGATATGGAC |  |
| *CAT* | -1.26431 | 0.000275 | AATCCGATAGGAGACAAAC | 164 |
|  |  |  | ACCTCAAAGTAGCCAAAA |  |
| *PPARA* | 2.278345 | 9.78E-07 | AGCGTGGCACTGAACATC | 143 |
|  |  |  | CTCCGATCACATTTGTCATAGA |  |
| *UCP3* | -1.7557 | 0.000232 | TGGGGTGGAGCCCTGAG | 193 |
|  |  |  | GCAGGGAGGTTCCAGGAGA |  |
| *PDK4* | -4.01542 | 0.000967 | CGGATGCTGATGAACCAACAC | 106 |
|  |  |  | GGACCACTGCTGCCACAT |  |
| *CEBPA* | 1.821306 | 0.004614 | GGCTACCTGGACGGCAGG | 187 |
|  |  |  | GCGGAGGGTGTGAATGCG |  |
| *SLC25A20* | -1.45768 | 0.001142 | GGAAACCAAGTACAATGGTG | 198 |
|  |  |  | ACTCATATGTCATGAAATAC |  |
| *EGFR* | 1.171987 | 0.002892 | ATCTGTAACCCGCTGTGCTC | 147 |
|  |  |  | GGCATTCTCCACGAACTCTC |  |

# log2Fold-Change and q-value are obtained from the RNAseq data
